# Current state and future of 3D bioprinted models for cardiovascular research and drug development

**DOI:** 10.5599/admet.951

**Published:** 2021-08-25

**Authors:** Liudmila Polonchuk, Carmine Gentile

**Affiliations:** 1 Pharmaceutical Sciences, Pharma Research and Early Development, Roche Innovation Center Basel, F. Hoffmann-La Roche Ltd., Basel, Switzerland; 2 Sydney Medical School, The University of Sydney, Australia; 3 School of Biomedical Engineering, University of Technology Sydney, Australia

**Keywords:** Bioengineered heart tissues, drug development, advanced *in vitro* models

## Abstract

In the last decade, 3D bioprinting technology has emerged as an innovative tissue engineering approach for regenerative medicine and drug development. This article aims at providing an overview about the most commonly used bioengineered tissues, focusing on 3D bioprinted cardiac cells and how they have been utilized for drug discovery and development. The review describes that, while this field is still developing, cardiovascular research may benefit from laboratory-engineered heart tissues built of specific cell types with precise 3D architecture mimicking the native cardiac microenvironment. It also describes the role played by regulatory agencies and potential commercialization pathways for direct translation from the bench to the bedside of studies using 3D bioprinted cardiac tissues.

## Introduction

Cardiovascular disease (CVD) is a leading cause of death worldwide, especially in the aging population. The increased incidence of CVD has been more recently associated with co-morbidity with other chronic diseases, such as kidney failure and type II diabetes, accounting for nearly one third of all deaths [1-4].

Over the past three decades extensive exploration in cardiovascular research to both prevent and treat CVD in patients has resulted in improved survival rates and quality of life [3,5]. Commonly practiced therapeutic interventions include drugs and surgical procedures, which have allowed a significant reduction in the mortality of CVD patients, followed by lifestyle changes to complement these primary interventions [2,5]. However, the lack of translation from the bench to the bedside, caused by limitations of currently available *in vitro* and *in vivo* models, has prompted a need to engineer novel tools for cardiovascular research. In the past two decades, 3D bioprinting technology has emerged as a potential tool to address limitations of conventional models by generating complex multicellular systems using cells and biomaterials that better recapitulate the human heart microenvironment [4,6,7]. More recently, together with the use of stem cells, advances in the field of biofabrication enabled a move toward advanced *in vitro* models for drug testing. This brief review aims to explore how cardiac bioprinting is best used for development of predictive *in vitro* tools to speed up development of new medicines and cell therapies, by covering technical and regulatory aspects of this technology.

## Cardiac Cells in 3D

Three-dimensional cell cultures and technologies are rapidly gaining recognition for their potential to model heart tissue pathophysiology [4,8-11]. Cells can be grown in scaffolds, scaffold-free or matrices environment aiming to mimic the ECM features of the heart; for example, biomaterial scaffolds, such as collagen and fibrin, provide a 3D environment for cells to attach, interact with each other and conduct electrical signals [4,6,7,12]. Cardiac myocytes cultured in 3D often employ a biomaterial such as a hydrogel or biocompatible polymer to mimic the ECM, providing a 3D architecture for cells to interact in all spatial dimensions, both with other cells and their environment. The ability to modify properties such as elasticity, stiffness, conductivity and porosity allows for fine-tuning of the microenvironment [6,12]. These are core aspects of cardiac tissue engineering as the utility of 3D culturing and bioengineering to simulate blood flow, observe contractile forces and relaxation velocity in cardiac myocytes with variable mechanical and electrical cues are the tools necessary to create a complex and accurate microenvironment [6,12,13]. The increase in parameters brings increased complexity and lack of standardized 3D culture protocols results in customized experimental design, which is not compatible with a high-throughput testing.

In a recent publication by Campostrini *et.al.* [14], a comprehensive overview of published 3D cardiac models was provided. Historically, engineered heart tissues (EHTs) have been used as a first approach to replicate the complex human heart microenvironment using patient-specific hiPSC-CMs to evaluate mutations, drug screening and individual risk of a patient such as drug-induced side effects [15,16]. Given their three-dimensional nature, EHTs allow the measurement of cell contractility in pathophysiological conditions together with measurements of contraction kinetics, rhythm and rate, genetic and protein analyses, and histological analyses of semi-thin, paraffin or ultrathin sections [17,18]. Another popular experimental approach of culturing cardiac cells in spheroids can be applied in several ways, both with and without a scaffold to support the spheroid development in a hanging drop or in low surface adhesion plates [19,20]. A cell-sheets technique utilizes temperature-sensitive surfaces to culture monolayers of cells that can be detached as a sheet of cells and continuously stacked over each other, resulting in a thick sheet of cells [21]. More recently, 3D bioprinting of cardiac tissues emerged as improved *in vitro* models of human heart pathophysiology [7].

## 3D Bioprinting of Cardiovascular Tissues

**Figure 1.**
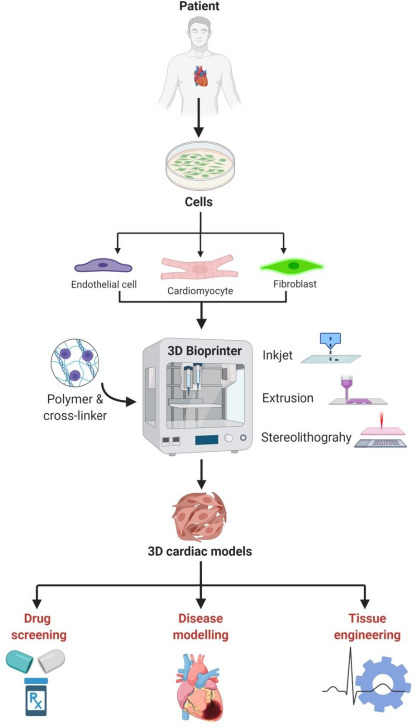
3D bioprinting technology and its applications in biomedical research.

Various processes have been applied for bioprinting cardiac constructs (Figure 1). Biofabrication of cardiac tissues using 3D bioprinting technology has approach-dependent advantages and disadvantages [22]. 3D bioprinting of heart tissues combines additive manufacturing, biomaterials and viable objects to produce structured biological constructs which can be assembled and oriented. This new approach enables designed anisotropy for directed response, controls 3D cell composition and alignment mimicking native myocardium [4,6].

Extrusion-based bioprinters dispense cells embedded in hydrogels continuously in a pre-defined shape using either pneumatic or mechanical forces. This technique is the most common and the least expensive. It allows rapid printing times and can print extremely high cell densities. However, material choice is limited as viscous bioinks are required for optimal extrusion through the nozzle, a feature that can affect cell viability. For instance, Kolesky *et al.* [23] bioprinted preformed vascular networks by lining human umbilical vein endothelial cells (HUVECs) viable for at least 6 weeks. Inkjet bioprinting allows the release of fluid droplets at precise locations via thermal or piezoelectric forces. This method yields high print resolutions as low as 20 μm, is compatible with a large range of bioinks and results in cell viability that can vary depending on the pressure applied. If low pressures are used, delicate cell types can be used, but this comes at the cost of lower structural integrity and, therefore, lower printed cell densities. Xu *et al.* [24] used primary feline and H1 cardiomyocytes on a controlled porosity alginate hydrogel to bioprint viable cell populations, indicating inkjet bioprinting may be useful in engineering designed cardiac tissues. 3D bioprinting using pre-formed cardiac spheroid cultures has been employed in an effort to use microtissues as building blocks, by stacking each individual spheroid in a needle-based array, free from any hydrogel addition [25]. In stereolithography, unlike the previous methods, the construct is hardened *via* photopolymerization from a vessel of fluid containing photoactive polymers. This method is rapid and removes physical stress on cells and bioinks resulting in moderately high resolutions down to 50 μm and high cell viability. However, the number of available photoactive polymer materials is limited, and their use in cell culture is further restricted by an application of ultraviolet light harmful for the cells [26]. Stereolithography bioprinting has mainly been used to generate patient-specific models to assist in surgical planning and to create vascularised tissue constructs from photoactive polymers with modifiable elasticity and tensile strength [26]. General schematics of the 3D bioprinting technology, and its application in biomedical field, are shown in Figure 1.

Bioprinted cardiac tissues have been generated to mimic several features of the cardiac microenvironment for both *in vitro* and *in vivo* applications. These include modelling for complex diseases, drug screening and potential transplantation to replace or support the regeneration of damaged myocardium. The vascularization of the cardiac tissue, which poses a major problem for long-term survival of cells in bioengineered tissues, has progressed with the advent of bioprinting. This includes generation of vascular networks through several methods including: (i) vascular structures via simultaneous bioprinting of cells and biomaterials; (ii) addition of angiogenic factors in bioprinted constructs; and (iii) bioprinting of channel-based constructs for pre-fabricated vascular networks. Previously generated spheroids have been bioprinted, used as building blocks and subsequently fused into vascular constructs with a range of cell types including human smooth muscle cells, human dermal fibroblasts and more importantly cardiac fibroblasts, endothelial cells (ECs) and iPSC-CMs [4,6,7,23,25]. Inkjet based bioprinters can be utilised to deposit biomaterial scaffolds and ECs simultaneously to form microvasculature scaffolds allowing EC proliferation into tubular structures with clinically relevant cell viabilities and maintained structural integrity of vasculature [27,28]. Angiogenic growth factors have been explored in bioprinted constructs with some success. HUVECs cultured in VEGF before bioprinting with iPSC-CM were reported to integrate with host vasculature when transplanted in mice [29]. Furthermore, VEGF slowly released into scaffolds (demonstrated with both Matrigel and alginate) promotes vessel formation and CD31 expression, which similarly have seen promising results in mice transplants [30]. More physiological constructs require a vascular network that supports flow throughout the entire structure. Bioprinting uniquely offers this feature of manufacturing complex and organized networks to enable nutrient delivery for the efficient cardiac function, waste and oxygen transport, all aimed at promoting cell survival and function [4,7]. This can be accomplished by bioprinting a hydrogel containing a removable internal structure, such as sacrificial polymer or spheroids, yielding hollow networks that can be populated with ECs to mimic *in vivo* vasculature [31-33]. In particular, the FRESH method allows integration of thermosensitive hydrogels such as gelatin and Pluronics for the generation of hollow structures [34]. Additionally, direct bioprinting of perfusable constructs is possible using multiple print-heads containing an outer cross-linkable hydrogel (*e.g.*, GelMA) and an inner head with the appropriate cross-linking solution [35].

Despite the latest improvements in structural organization of cells within bioprinted cardiac tissues, their optimal vascular network and contractile function remain the major challenges to overcome before expanded use of clinically relevant constructs could be readily available [4]. Cardiac spheroids from iPSC-CMs already have been demonstrated to spontaneously contract but are limited by immature phenotypes [9,20,36]. Therefore, multitude efforts seek to improve the microenvironment of iPSC-CMs and the tissues they are grown within to increase cell contractility and contribute to overall cardiac tissue development [37]. Among these strategies, the cardiac environment has been improved by the addition of conductive polymers for electrical propagation support and elastic polymers for mechanical support [38-40]. Though these biomaterials have been used in cardiac tissue engineering previously, optimizing such biomaterials for bioprinting is feasible and slowly progressing, yet still requires further studies to improve the effects on biocompatibility [4].

## Application of 3D bioprinting in cardiac regeneration and drug testing

The heart has a limited capacity to regenerate after birth, and the major focus of cardiac bioprinting has been the generation of tissues that may represent an alternative approach to regenerate infarcted heart tissue by integrating cardiac cells in presence of additional biomaterials [4,41]. The application of bioprinted cardiac tissues potentiates the creation of functional cardiac tissue to regenerate or replaced damaged tissue in the myocardium [37,41,42]. In a similar effort to the one used by other approaches aimed at bioengineering the cardiac tissue, these bioprinted tissues better mimic structural, physiological, and functional features of the native myocardium, all features that can be applied for drug testing, toxicity assays and disease modelling (*e.g.*, myocardial infarction and heart failure [7,29,43]). This approach allows the generation of a complex 3D structure permissive for tightly-regulated molecular and cellular interactions, by supporting cells and enhancing their structural reorganisation into functional cardiac tissues [44]. Optimal *in vitro* maturation and functional testing (including measurements of durability of tissues and their contractile function) before bioprinted tissues can be transplanted onto the defected heart is critical [4,6]. Among the several tests, cell viability assays, vascular network evaluation and optical-electrical mapping tests are required for quality control and to characterize their structural, mechanical and electrical properties [10,25,29,45].

Bioprinting holds promise as a potential clinical tool to enable production of vascularized tissues with nutrient, waste and oxygen transport [46]. For example, Jang *et al.* [47] developed a pre-vascularized 3D bioprinted myocardial tissue using bioinks containing either cardiac progenitor cells (CPCs) or human dermal tissue-derived microvascular endothelial cells (ECs) together with turbinate mesenchymal stem cells (MSCs). Cells were dispersed in a human decellularized extracellular matrix (dECM) within the bioinks. In this study, the two bioinks were used following an alternative patterning design as dual cell threads, which facilitated the vascularization of the patch, as tested in an *in vivo* rat model of heart failure. However, in this study a branched endothelial cell network typical of the human myocardial tissue could not be established, remaining one of the major challenges for the 3D bioprinting of viable and functional cardiac tissues [4].

In a study by Mirdamadi *et al.* [48] using the FRESH printing technique described above, whole-size heart constructs were 3D printed by embedding soft biomaterials in a thermoreversible support bath. This study expanded the printable size range by FRESH printing to a full-size model of an adult human heart from patient-derived magnetic resonance imaging (MRI) data sets. Alginate hydrogels were used as the printing biomaterial to mimic the elastic modulus of the human cardiac tissue. FRESH-printed alginate proved to be a high print fidelity low-cost platform to create customized models for surgical training and planning. Future studies including cells will be required to demonstrate the feasibility of this approach for cardiac tissue engineering purposes.

Even when not 3D bioprinted, cardiac tissues derived from hiPSC-CMs embedded with extracellular matrix proteins (*e.g.*, fibronectin and gelatin nanofilm) present the potential to be used as a screening system for drug discovery and cardiotoxicity assay [49]. However, in addition to biological factors, a major challenge of *in vitro* cardiac tissues is their ability to respond to mechanical and electrical simulations in 3D [28]. In our latest study, we developed a novel approach for the biofabrication of 3D bioprinted heart tissues using bioinks containing alginate/gelatin (Al/Ge) hydrogels and 3D cardiac spheroids (CSs) [50]. Cardiac spheroids were first created from human cardiac myocytes, fibroblasts and endothelial cells and then mixed within optimal Al/Ge hydrogels before being tested for viability and contractile function *in vitro*. When 3D bioprinted in a CardioExcyte 96-well microelectrode plate, the response of 3D bioprinted CSs to isoproterenol treatment was recorded. Al/Ge hydrogels enabled CS adherence to the electrode and fusion, which was further facilitated by addition of vascular endothelial growth factor (VEGF). Bioprinted CSs contracted spontaneously and under electrical stimulation, allowing us to record contractile and electrical signals on the microelectrode plates. The ability of bioprinted CSs to respond to drug treatments in 3D makes them suitable candidates as high-throughput assays for industrial applications.

Another approach to vascularize 3D tissues includes the use of microfluidic “*organ-on-a-chip*” devices, which provide better control over the microenvironment by mimicking various *in vivo* mechanical and chemical functions of the heart and can be used to monitor parameters, such as pH, nutrient supply and oxygen level [28]. Microfluidics devices structure and maintain cellular morphology and cell-specific functionality of the 3D biofabricated tissue, while regulating shear stress forces through the flow rate [51]. In particular, they have also been used to promote endothelial cell network for optimal cell viability and function [52]. Undoubtably, organ-on-a-chip models have advanced our way of using cardiac cells *in vitro*, in a way to improve their use for drug screening and to improve treatment efficacy. However, more research is required to bioengineer a device that fully recapitulates the microenvironment typical of the cardiac tissue, including combinations of 3D bioprinting and microfluidics devices.

While the use of 3D cardiac bioprinting for direct clinical applications remains at an experimental stage, there are signs that the technology is slowly progressing towards more end-use applications in development of advanced cellular models for drug testing. Current regulatory mandated assays for cardiac safety either do not capture the complexity of drug-induced cardiotoxicity or as with *in vivo* animal studies cannot be extrapolated to humans. Consequently, the prediction of drug-induced cardiotoxicity remains a constant challenge for pharma-industry, and accounts for up to 33 % of drug failure due to drug withdrawal during clinical development [53]. In an effort to make cardiotoxicity testing more biologically-relevant and therefore translatable, the US Food and Drug Administration (FDA)-sponsored Cardiac Safety Research Consortium and the not-for-profit Health and Environmental Sciences Institute (HESI) have recommended incorporating hiPSC-CMs into the nonclinical drug cardiotoxicity assessment as an integrating model system bridging single receptor-based *in vitro* results together with clinical data [54]. The strategy that integrates human iPS-cardiomyocytes engineered into a 3D system mimicking cardiac microenvironment offers an opportunity to develop robust, physiologically relevant *in vitro* functional assays, with the potential to better predict clinical outcomes, reduce animal usage, speed up decision making and lower development costs.

## Engagement of public private partnerships for transition from the bench to the bedside

**Figure 2.**
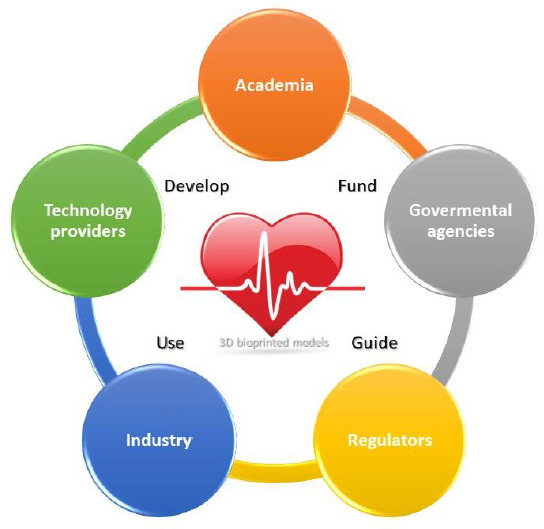
*Stakeholder’s communication scheme*. Schematic showing key roles and how information flow and relationships contribute to new model development.

In the process of generating more physiologically relevant *in vitro* models, regulatory agencies are preparing for ramifications of biofabricated tissue products (Figure 2). However, current FDA guidance primarily addresses technical aspects of devices used for 3D printing methods and products [55]. Lack of regulatory standards makes it difficult to commercialize novel bioprinting technologies. Public acceptance of *in vitro* 3D bioprinted models needs to be preceded by regulatory policies that guide technology companies within the field. Ideally, they should work closely with regulatory agencies and potential customers to ensure product validation for industrial use. Eventually, the purpose and the mechanistic question will determine the model to be utilized for specific applications. A recent FDA white paper co-authored by academic and industrial experts outlined the general validation principles for the models to be used in the field of cardiac safety [56].

Advances in the regulatory process may significantly incentivize maturation and commercialization of 3D bioprinting research in general. And government organizations could foster advancements in fundamental bioprinting technologies and potentially support early investments in this field. They can help demonstrating the utility and disseminate the use of this emerging technology into a variety of communities including academia, government, regulators, NGOs, and industry. Well-balanced interaction between all the stakeholders, schematically illustrated in Figure 2 is essential to assure progress and foster growth on a global scale. Agencies such as the US National Science Foundation (NSF) and Department of Defense (DoD) awarded multiple grants to institutions dedicated to studying and developing biomaterials specifically for use in bioprinting applications over the past decade.

Big healthcare companies followed the trend and set up collaborations with bioprinting companies. For instance, AstraZeneca has established the BioVentureHub, which provides emerging life science companies and academic teams access to AstraZeneca’s experience and infrastructure. CELLINK, a global leader in bioprinting, has already established an R&D lab in BioVentureHub to biofabricate kidney, heart, liver, and lung tissue models for pharmaceutical development. Likewise, J&J announced the creation of a bioprinting research laboratory as part of the 3D Printing Center of Excellence in collaboration with the Advanced Materials and BioEngineering Research (AMBER) Institute in Dublin. Astellas Pharma, Bristol-Meyers Squibb, Merck, Novartis, Procter and Gamble, Roche also have bioprinting programs as do some large research facilities around the world, such as the National Institutes of Health (NIH) in the US. In Australia, despite the lack of big national pharmaceutical companies, there has been a focus on emerging bioprinting technologies, with several centers supported by the government (i.e., the ARC Training Centre in Wollongong, NSW, the ARC Centre of Excellence for Electromaterial Science). The emerging interest in this field is also highlighted by the recent Bioprinting Workshops organised by the University of Technology Sydney (UTS) in partnerships with global industry partners [57]. Abovementioned workshops and platforms for continuous education and information sharing may represent the first step in effectively bringing together future partners and catalysing public-private partnerships [57]. In the long run, innovation labs and start up incubators in commercial and public healthcare sectors would be the most effective way to develop the research idea into a commercial product. Therefore, an international network composed of experts from academia, industry and regulatory bodies should facilitate the frameworks to promote fast and efficient bench-to-bedside technology transfer.

## Conclusions and future directions

Emerging 3D bioprinting technologies offer therapeutic strategies to better combat heart diseases, as well as open the door for improved drug testing models in drug development. They hold much promise in personalized and regenerative medicine, particularly with the opportunity of using patient-specific cells. They also uniquely allow to recapitulate the 3D cardiac tissue architecture required for functional human myocardium. Development of cardiac-specific bioinks and efficient printing methods for high throughput assays would enable the fabrication of large-sized engineered tissue models. A faster adoption, commercialization and validation of 3D bioprinted cardiac tissues will depend on coherent strategic partnerships between academia, regulators and industry and will benefit patients and society.
